# Supplementary material for: Medical service quality, patient satisfaction and intent to revisit: Case study of public hub hospitals in the Republic of Korea
Source: PLoS One. 2021 Jun 28;16(6):e0252241. doi: 10.1371/journal.pone.0252241 (PMC8238216; doi:10.1371/journal.pone.0252241)
Supplement: Attachment — Submitted filename: Reviewers comments- RKK 02.03.21.docx [file pone.0252241.s001.docx]

**Title: The effect of quality of medical service on patient satisfaction and intent to revisit: for public hub hospitals in Republic of Korea**

**(Date: 17.01.2021)**

**Authors:** Selin Woo and Mankyu Choi

**Reviewer’s Comments:**

The paper under review is very good and conducted in scientific manner. The authors have statistically analyzed the causal relationship between quality of healthcare at public hub hospitals, patients’ satisfaction, and their intension to revisit the same hospital in future. The study aimed to provide fundamental data to improve patient satisfaction and service in regional medical centers and Red Cross hospitals. For this the data on both outpatients and inpatients were taken from the “2018 Regional Hub Public Hospital Operational Evaluation”, conducted by the Ministry of Health and Welfare and the National Medical Center. A number of analysis and structural equation model were used to understand the relationship between patient satisfaction and intent to revisit.

The study has concluded the following:

- **In the direct effect on the outpatient category**, the better the physician's practice service, physical environment of the hospital, and patient satisfaction, the more likely there were to increase the patients’ intent to revisit. **The indirect effect** on intent to revisit through patient satisfaction was highest for physician's practice service, followed by medical staff's kindness and consideration, and physical environment of the hospital.
- **In the direct effect on the inpatient category of patients**, better physician's practice service, medical staff's kindness and consideration, and patient satisfaction were likely to increase the patients’ intent to revisit and **the indirect effect** on intent to revisit, through patient satisfaction was highest for medical staff's kindness and consideration, then nurse's practice service, physician's practice service, and patient satisfaction.

The authors have also given a few suggestions at the end of the paper based on the results and analysis. The paper is successful in its attempt and addressed its objectives well. However, it may also shed lights on some other issues related to patient’s satisfaction. This may not be necessarily related to the quality of healthcare as perceived by the patients, such as some local issues related to the local culture and tradition, practices, etc. Issues of privacy and confidentiality, counselling by doctors, nurses or designated, especially for the inpatients suffering from some major ailments, etc. are also major factors which affect the satisfaction of the patients and their intension to revisit the public health facilities. The problem of overcrowding and shortfalls of medical and paramedical staff especially in the developing country is also a major challenge faced by the public health facilities.

Rajeev Kamal Kumar

(Ph.D. Anthropology)

A N Sinha Institute of Social Studies, Patna
